# Assessment of occupational injury and associated factors among Awash Wine Beverage Factory workers in Addis Ababa, Ethiopia

**DOI:** 10.1097/MD.0000000000049423

**Published:** 2026-06-26

**Authors:** Mequanente Dagnaw, Meera Indracanti, Yordanos Kinfe, S. Hari Priya, Suleyman Mohammed Arage

**Affiliations:** a Department of Epidemiology and Biostatistics, Institute of Public Health, University of Gondar, Gondar, Ethiopia; b Department of Medical Biotechnology, Institute of Biotechnology, University of Gondar, Gondar, Ethiopia; c Department of Medical Biotechnology, School of Allied and Healthcare Sciences, Malla Reddy University, Hyderabad, Telangana, India; d Department of Public Health, Ayer Tena Health Science and Business College, Addis Abeba, Ethiopia; e Department of Biochemistry, School of Allied and Healthcare Sciences, Malla Reddy University, Hyderabad, Telangana, India; f Department of Public Health, College of Medicine and Health Science, Werabe University, Worabe, Ethiopia; g Department of Health System and Policy, Institute of Public Health, College of Medicine and Health Science, University of Gondar, Gondar, Ethiopia.

**Keywords:** Addis Ababa, Awash Wine Beverage Factory, beverage factory, occupational injury

## Abstract

Occupational injury is a major public health and workplace safety problem in manufacturing industries. In Ethiopia, the magnitude of occupational injury among beverage factory workers ranges from 20.9% to 44.66%. Uncontrolled occupational injuries can lead to permanent disability, reduced work capacity, psychological stress, loss of income, decreased productivity, and death. These injuries also impose economic burdens on workers, families, industries, and the national healthcare system and negatively affect quality of life and community stability. This study aimed to assess the magnitude of occupational injury and its associated factors among workers at the Awash Wine Beverage Factory in Addis Ababa, Ethiopia, in 2024. An institution-based cross-sectional study was conducted among 279 randomly selected workers from 3 branches of the Awash Wine Beverage Factory. Data were collected using a structured interviewer-administered questionnaire and a workplace observation checklist. Data were entered into EpiData 3.1 and analyzed using STATA 15.1 (Stata Corp LLC). Chi-square tests and logistic regression analyses were performed. Variables with *P* < .25 in bivariate analysis were included in multivariable logistic regression. A *P*-value of <.05 at a 95% confidence interval (CI) was considered statistically significant. The prevalence of occupational injury within the previous 12 months was 43 (15.9%; 95% CI: 11.8%–19.9%), while injuries in the 2 weeks before data collection were 14 (5.2%; 95% CI: 2.6%–8.1%). Workers with ≤5 years of work experience were more likely to sustain injuries (adjusted odds ratio [AOR] = 5.86; 95% CI: 1.72–20.10). Not using personal protective equipment (PPE; AOR = 2.06; 95% CI: 1.02–2.23) and working at heights (AOR = 8.69; 95% CI: 5.94–8.85) were also more likely to sustain injuries. In addition, lack of regular health and safety supervision was significantly associated with injury (AOR = 5.9; 95% CI: 1.77–19.62). Occupational injury remains a serious concern among Awash Wine Beverage Factory workers. Short work experience, lack of PPE use, unsafe working conditions, and poor safety supervision were significantly associated variables. Strengthening safety training, improving PPE utilization, enhancing workplace conditions, and ensuring regular supervision are essential to reduce injuries and improve worker safety and productivity.

## 1. Introduction

Occupational injury is any personal injury that can lead to disease, disability, or death due to injuries sustained by workers while performing their work.^[1]^ The workplace is a potentially hazardous environment where several employees spend at least one‐third of their day. This might affect workers’ health and safety and could lead to various work-related injuries.^[2]^ An occupational injury is an unexpected and unplanned occurrence, including acts of violence, arising out of or in connection with work, which results in 1 or more workers incurring a personal injury, disease, or death.^[3]^ A safe and healthy working environment is a vital aspect of the food and beverage processing industry.^[4]^ A beverage is defined as a liquid that is specifically prepared for human consumption.^[5]^

The food and drink manufacturing industry comprises more than 30 different industries. These range from slaughterhouses, sugar refineries, and grain mills to malt manufacture and whiskey distilling. The combined injury rate for the food and drink industries is among the highest; however, injury rates vary considerably across the food and drink industries.^[6]^

The beverage manufacturing industry has 2 categories: alcoholic and nonalcoholic. The subgroups of the latter category encompass soft drinks, water bottling, fruit juice bottling, canning, boxing, the coffee industry, and the tea industry. In contrast, the former subgroups are distilled spirits, wine, and brewing.^[7]^ The beverage sector remains the largest industrial manufacturing sector in most European Union countries, contributing more than €1.109 trillion in annual turnover and employing more than 4.57 million people.^[8]^

The most commonly encountered injuries in beverage and wine manufacturing are related to car accidents while transporting raw materials for production, the use of chemicals for sanitizing bottles, and wastewater treatment. Chemical exposure of employees and the environment, slippery floors, and work at height are among the factors that cause occupational injuries, as they increase employees’ risk of falls and other injuries.^[9]^

The brewing industry has many production workers and work-related problems that have yet to be explored independently in Ethiopia.^[9]^

According to the latest estimates, over 395 million workers worldwide sustained a nonfatal work injury in 2019. In addition, around 2.93 million workers died as a result of work-related factors, an increase of more than 12% compared with 2000 (International Labor Organization [ILO], forthcoming).^[10]^ Furthermore, according to the ILO, it is estimated that 2.78 million occupational fatalities and 374 million nonfatal occupational injuries and illnesses occur globally each year.^[11]^

Beyond fatalities, occupational injuries contribute to the overall impact on global health and development, as the human cost and economic burden of poor occupational health and safety (OHS) practices are estimated to amount to a loss of up to 6% of gross domestic product in some countries.^[10]^ Evidence shows that the direct cost of a workplace injury can account for 14% to 16% of payroll expenses, and the indirect cost can account for 42% to 48%.^[12]^

Worldwide, each year, 160 million people live with work-related injuries, resulting in 4 or more days of work absence.^[13,14]^ According to the ILO report on the 20th World Congress, the estimated average cost of occupational injuries is 4% of global GDP. Furthermore, about 19% of deaths attributed to work are due to occupational injuries.^[13]^ The prevalence of occupational injuries among beverage factory workers in Ethiopia ranges from 20.9% to 44.66%.^[9,15]^

Regarding the severity of occupational injuries, their occurrence, if not controlled, can cause fatalities and disabilities and reduce productivity. It also increases compensation-related costs.^[16]^ A study assessing the epidemiology of occupational injuries in the Ethiopian hotel industry found that approximately three-fourths (74.4%) of injured hotel workers were hospitalized, with 26.1% staying in the hospital for up to 5 days. Similarly, more than three-fourths (76.6%) of injured workers took paid sick leave. As a result of the injury, a total of 1015 working days were lost over the last 12 months, averaging 1 day per injured worker. Furthermore, it showed that 41.6% of respondents had disabilities, of which 96.2% and 3.8% were temporary and permanent types of disability, respectively.^[17]^

The ILO is committed to improving the health and safety of workers, which it achieves by controlling workplace hazards and injuries and by complying with strict legal requirements. As a result, most organizations have implemented workplace safety management systems through documentation and certification to minimize injuries.^[18]^ Over the past 20 years, Ethiopia has strengthened its application of OHS measures to mitigate workplace hazards. Labor unions have also been successful in improving working conditions. Nonetheless, there are still health concerns related to solid waste management that need to be addressed.^[19]^

Scarcity of resources, lack of well-equipped facilities, personnel shortages, overcrowding, lack of training, and improper execution of safety guidelines and regulations are key factors that increase the risk of occupational injuries among wine factory workers.^[11,20–22]^

## 2. Methods

### 2.1. Study area and period

The study was conducted from July 15 to July 30, 2024, at the Awash Wine Beverage Factory in Addis Ababa, Ethiopia. In Ethiopia, 11 brewery plants were owned by 7 firms. Currently, following a privatization policy, all 11 plants are privately owned. The Awash Wine Beverage Factory was one of the privately owned wine beverage factories in Ethiopia. It has a tradition of winemaking that stretches unbroken to the region of Queen Sheba beyond 2 Greek and Italian families. In 1936, a Greek family started the first winery in Ethiopia in Addis Ababa at Lideta, followed by an Italian family who established their winery at Mekanisa. The 2 ventures were nationalized in 1974 and regrouped into a single entity named Awash Wine. Since then, Awash Wine has grown into a household name and one of Ethiopia’s most loved brands, a market leader interwoven with the cultural fabric of society. It is located in Addis Ababa. There were around 1040 workers in this company.

### 2.2. Study design

An institution-based cross-sectional study was conducted.

### 2.3. Population

#### 2.3.1. Source population

All workers employed at the Awash Wine Beverage Factory.

#### 2.3.2. Study population

All workers employed at the Awash Wine Beverage Factory during the study period, except administrative staff such as managers, finance individuals, and supervisors.

#### 2.3.3. Sample population

Randomly selected workers at the Awash Wine Beverage Factory who were employed during the study period and met the inclusion criteria.

### 2.4. Inclusion and exclusion criteria

#### 2.4.1. Inclusion criteria

All workers working in the Awash Wine Beverage Factory during the study period.

#### 2.4.2. Exclusion criteria

Those workers who did not have direct contact, such as administrative staff (managers, finance individuals, and supervisors), and workers who were on annual leave or absent during the data collection period, as well as temporary workers, subcontracted labor, or seasonal employees, were excluded since they did not have similar exposure structures.

### 2.5. Sample size and sampling procedures

#### 2.5.1. Sample size determination

The sample size was determined using the single population proportion formula. The following assumptions were used for 2 specific objectives: a confidence interval (CI) of 95% with a margin of error (*d*) of 5%, a design effect of 5, and the magnitude of occupational injury among wine beverage factories for both objectives as 20.9% from a previous study,^[9]^ and a 10% nonresponse rate, n = 254. After adding a 10% nonresponse rate, the sample size was 279 workers. Although simple random sampling was employed at the individual level, a design effect of 5 was applied during sample size estimation to account for anticipated nonindependence of observations due to shared work environments. Workers were drawn from multiple departments and shifts where exposure to similar machinery, safety practices, and supervision could introduce intragroup correlation in occupational injury occurrence. Previous occupational health studies have reported moderate intraclass correlation coefficients for injury outcomes within work units, which can substantially inflate variance. Therefore, a conservative design effect was applied to ensure adequate statistical precision and to minimize the risk of underestimating the required sample size.

#### 2.5.2. Sampling procedures/techniques

Employees of the Awash Wine Beverage Factory were selected from the official employee roster obtained from the Human Resources Department. This list encompassed all permanent and contract employees who were actively working at the time of data collection. The roster functioned as the sampling frame.

To ensure the list’s accuracy, the researchers verified the roster against attendance records and consulted departmental supervisors to confirm that only currently active employees were included. Those on long-term leave, sick leave, or temporarily assigned outside the factory during the data collection period were excluded from the sampling frame.

Study participants were subsequently selected using simple random sampling based on the verified list. Each employee was given a unique identification number, and random numbers were generated to determine the necessary sample size.

In instances where selected employees were unavailable after 2 attempts to visit or chose not to participate, replacements were sourced from the remaining employees in the sampling frame using the same random selection technique. This replacement process was implemented to preserve the original sample size and minimize sampling bias. Although simple random sampling was employed, a conservative inflation factor was applied during sample size estimation to account for potential heterogeneity in working conditions across job categories and to minimize the risk of underpowering. This approach was used for planning purposes only and does not imply a clustered sampling design.

### 2.6. Study variables

#### 2.6.1. Dependent variable

Occupational injury.

#### 2.6.2. Independent variable

Sociodemographic factors: sex, age, marital status, religion, educational status, work experience, monthly salary, and employment type.

Organizational (working environment) factors: health and safety training, safety signs, job category, working hours per day, and supervision.

Utilization of personal protective equipment (PPE): availability of PPE and accessibility of PPE.

Behavioral factors: drinking alcohol, smoking cigarettes, chewing khat, sleep problems, job satisfaction, job stress, and use of PPE.

Knowledge of safety precautions: **when to use standard precautions, how to use a safety container**, and how to use PPE.

### 2.7. Operational definitions

Lifetime occupational injury: Work-related injuries that occur on the job and are a direct result of the tasks allotted to the specific job.^[9]^

Occupational injury in 12 months: Work-related injuries that occurred on the job and were a direct result of the tasks allotted to the specific job 12 months before the study, excluding chronic exposure. Respondents were asked whether they had an injury within 12 months; those who answered “yes” were considered to have an occupational injury within that period.^[9]^

Occupational injury in 1 month: Work-related injuries that occurred on the job and were a direct result of the tasks allotted to the specific job in the 1 month before the study; chronic exposure was excluded. Respondents were asked whether they had an injury within the past month, and those who answered “yes” were considered to have an occupational injury in that month.^[9]^

### 2.8. Data collection tools and procedure

#### 2.8.1. Data collection tool

A structured, interviewer-administered questionnaire was used. The questionnaire included sociodemographic characteristics, working environmental factors, behavioral factors, the utilization and availability of PPE, and knowledge of safety precautions to assess the magnitude of occupational injuries and their determinants among workers at an Awash Wine Beverage Factory. The tools were developed by adapting variables from relevant literature. They were adopted and contextualized in line with the study objectives, and then translated from English into Amharic. The tool underwent translation/back-translation procedures, content or face validity assessment, and reliability testing using the Cronbach α test. The finding was α ≥ 0.89, indicating an excellent tool for data collection in Ethiopia.

#### 2.8.2. Data collection procedure

The data were collected through face-to-face interviews with 3 environmental health professionals under the supervision of 1 public health professional. The data were obtained at the institutional level after proportional allocation was made to each branch based on the number of workers in each branch. One data collector was assigned to each branch, and they provided details about the study before obtaining consent to participate. Participants who were willing to provide verbal or written informed consent were interviewed during a tea break in a quiet place. Inter-observer variability was reduced by implementing standardized training, utilizing uniform tools, conducting pretests, performing supervisory checks, and assessing inter-rater reliability. Interviewer bias was mitigated by keeping interviewers unaware of the study hypotheses, using neutral, standardized questions, and ensuring ongoing supervision during data collection. Occupational injury was assessed using a standardized, structured questionnaire with a clearly defined reference period for injury. Data collectors were trained to probe consistently to improve recall accuracy.

### 2.9. Data quality assurance

Data quality was ensured through careful design of data extraction formats, appropriate recruitment of data collectors, and adequate training and follow-up for data collectors and supervisors. The questionnaire was pretested on 5%^[14]^ of workers at the Heineken Wine Factory and was modified accordingly before the main study began. Advisors evaluated the face and content validity of the tool’s final version before and after the pretest. Two days of intensive training were given to data collectors and supervisors. The investigators and supervisors provided intensive supervision throughout the data collection period. To confirm the reliability of the data during data collection, the investigators reviewed a random sample of questionnaires, and supervisors conducted random cross-checks of their completeness, accuracy, and consistency. The data collectors, under the close supervision of the principal investigators and supervisors, collected the data. Remarks were given in the morning on how to eliminate or minimize errors and take corrective action. The data were checked for completeness and consistency, then coded, entered, and stored on the computer.

### 2.10. Data processing and analysis

Data were entered into EpiData version 3.1 (Epi Data Association) and then exported to SPSS version 27 (IBM Corporation) for analysis. The normality of continuous variables was assessed by evaluating histograms. Normally distributed data were presented as mean (standard deviation). Categorical variables were expressed as percentages, and differences in proportions were compared using the chi-squared test. Logistic regression analyses were used to identify factors associated with occupational injury. Bivariate logistic regression was conducted to select candidates for multivariate logistic regression, with *P*-values <.25 at 95% confidence. Then, candidate variables were entered into a multivariate logistic regression. A statistically significant association was declared at a *P*-value of <.05. Variables included in the multivariable logistic regression model were selected a priori based on previous occupational health literature and theoretical relevance to occupational injury, including age, sex, work experience, job category, and working conditions. Additional variables that demonstrated plausible associations with both the outcome and key exposures were included to improve model stability. Multicollinearity was assessed before model fitting. All variables were retained in the final model to control for potential confounding rather than relying solely on statistical significance.

### 2.11. Ethical clearance and informed consent

Ethical clearance for this study was obtained from the Institutional Review Board of the GT Technology College (Reference No. GTTC/18/08/2024). Informed consent was not obtained because the study was retrospective. To protect participants’ rights and privacy, strict measures were taken to maintain anonymity and confidentiality throughout the study. Participants were not required to provide any personally identifying information, such as names, phone numbers, or addresses. Each participant was assigned a unique identification code, and all data were recorded using these codes instead of personal identifiers. The institutional ethical review board of the GT Technology College in Gondar, Ethiopia, authorized the participants’ informed consent waiver. The study was conducted in accordance with the ethical principles outlined in the Declaration of Helsinki (2013 version).

## 3. Results

### 3.1. Sociodemographic characteristics

A total of 279 eligible Awash Wine Beverage Factory workers were approached in this study, and 271 participated, for a response rate of 97.1%. The mean age of participants was 38.92 (standard deviation 8.2) years. 120 (44.3%) were older than 40 years, and 91 (33.6%) were unmarried. Of the 271 workers, 157 (57.9%) were male. Regarding educational and employment patterns, 36 (13.3%) were illiterate, and 55 (20.3%) were contract workers. Regarding monthly income and experience, only 71 (26.2%) workers had a monthly income of <6210 birr, and 161 (59.4%) had at least 5 years of work experience (Table 1).

**Table 1:** Sociodemographic characteristics of Awash Wine Beverage Factory workers in Addis Ababa, Ethiopia.

Variables	Category	Frequency	Percent
Sex	Male	157	57.9
	Female	114	42.1
Age in years	25–29 yr	35	12.9
	30–34 yr	61	22.5
	35–39 yr	55	20.3
	40–44 yr	53	19.6
	45 and above yr	67	24.7
Marital status	Married	153	56.5
	Single	91	33.6
	Divorced	19	7.0
	Widowed	8	3.0
Educational status	Illiterate	36	13.3
	Read and write	32	11.8
	Primary^[1–8]^	72	26.6
	Secondary	100	36.9
	College and above	31	11.4
Employment pattern	Temporary	55	20.3
	Permanent	216	79.7
Monthly income	<6210	200	73.8
	≥6210	71	26.2
Work experience	<5 yr	110	40.6
	≥5 yr	161	59.4

### 3.2. Utilization of personal protective

Among 271 workers, three-fourths of the participants, 215 (79.3%), used PPE while working; however, 129 (60%) of them claimed that they used it all the time at work, and more than half of them, 116 (54%), said that the institution supplied them. For those who did not use PPE, the major reason is to save time. Regarding training, 85 (31.4%) and 39 (14.4%) had training on any occupational safety issues and on-the-job training on such issues, respectively (Table 2).

**Table 2:** Utilization of personal protective measures of Awash Wine Beverage Factory workers in Addis Ababa, Ethiopia.

Variables	Category	Frequency	Percent
Using PPE	No	56	20.7
	Yes	215	79.3
Use PPE all the time while working	No	86	40.0
	Yes	129	60.0
Reasons not to use safety equipment all the time	To save time	28	50.0
	Not aware of the risk	12	21.4
	Carelessness or negligence	12	21.4
	No access	4	8.2
Source PPE	It is supplied by an institution	116	54.0
	You buy it for yourself	89	41.4
	Others	10	4.6
Had training on any type of occupational safety issues	No	186	68.6
	Yes	85	31.4
Had on-the-job training on any type of occupational safety issues	No	232	85.6
	Yes	39	14.4

PPE = personal protective equipment.

### 3.3. Type of PPE used by workers

**Figure 1.**
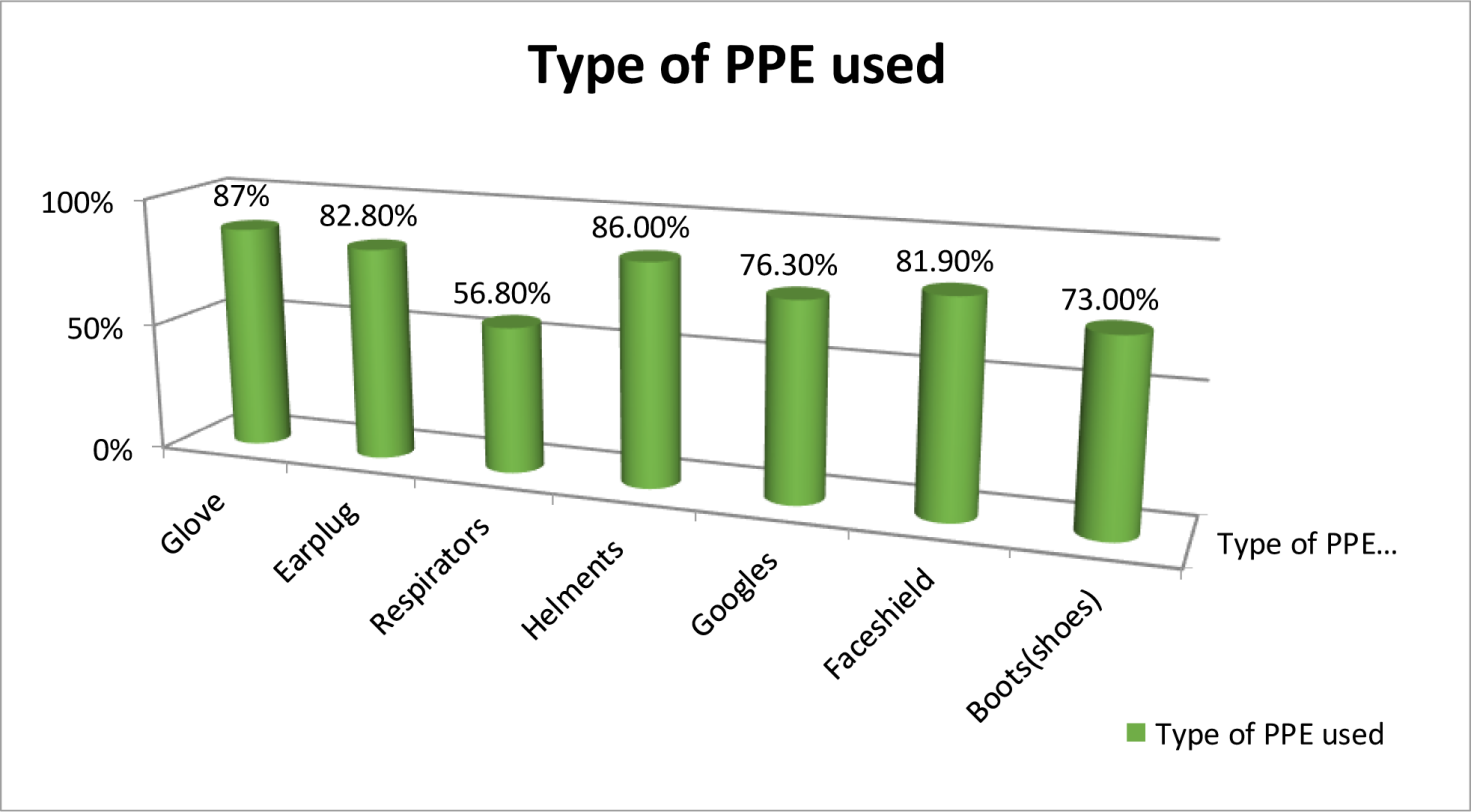
Type of personal protective equipment used by Awash Wine Beverage Factory workers in Addis Ababa, Ethiopia, 2024. PPE = personal protective equipment.

Regarding the type of PPE used by workers, gloves were the most commonly used PPE, while respirators were the least commonly used PPE by workers, as shown in the figure below (Fig. 1).

### 3.4. Occupational (work-related) injury characteristics

This study revealed that 43 (15.9%) of the Awash Wine Beverage Factory workers, with a 95% CI of 11.8% to 19.9%, had an injury at work within 12 months before the study period, and injuries 2 weeks before the study accounted for 14 (5.2%) with a 95% CI of (2.6–8.1). Twenty-seven (62.8%) of them had been injured with a frequency of 3 or more in 12 months. Regarding the type of injury, falls from height were the most common, reported by 50% of workers (21; 48.8%). The most common reason for time lost was being new to the work process, and the most commonly reported private affair was the death of a family member (Table 3). One-third (30%) of workers had a history of hospitalization.

**Table 3:** Occupational (work-related) injury characteristics among Awash Wine Beverage Factory workers in Addis Ababa, Ethiopia.

Variables	Category	Frequency	Percent
Had an incident at work that resulted in injury in the last 12 mo	No	228	84.1
	Yes	43	15.9
Had an incident at work that resulted in injury in the last 2 wk	No	257	94.8
	Yes	14	5.2
Frequency of injury times in the last 12 mo	<3	16	37.2
	≥3	27	62.8
Time of injury	Morning	16	37.2
	Afternoon	17	39.5
	Don’t know	10	23.3
Types of accidents	Falling from a height	21	48.8
	Struck by an object	10	23.3
	Hit by a falling object	6	14.0
	Falling at ground level	6	14.0
The reason at the time of injury	Being new to the work	15	34.9
	Thinking about private affairs	15	34.9
	The accident was beyond control	13	30.2
Working condition	At height	44	16.2
	Ground	162	59.8
	Both	15	5.5
	Underground	50	18.5
History of admission due to injury	No	23	53.5
	Yes	20	46.5
Duration of hospitalization in the day	≤1 d	14	70.0
	>1 d	6	30.0

### 3.5. Parts of the body affected by the injury

**Figure 2.**
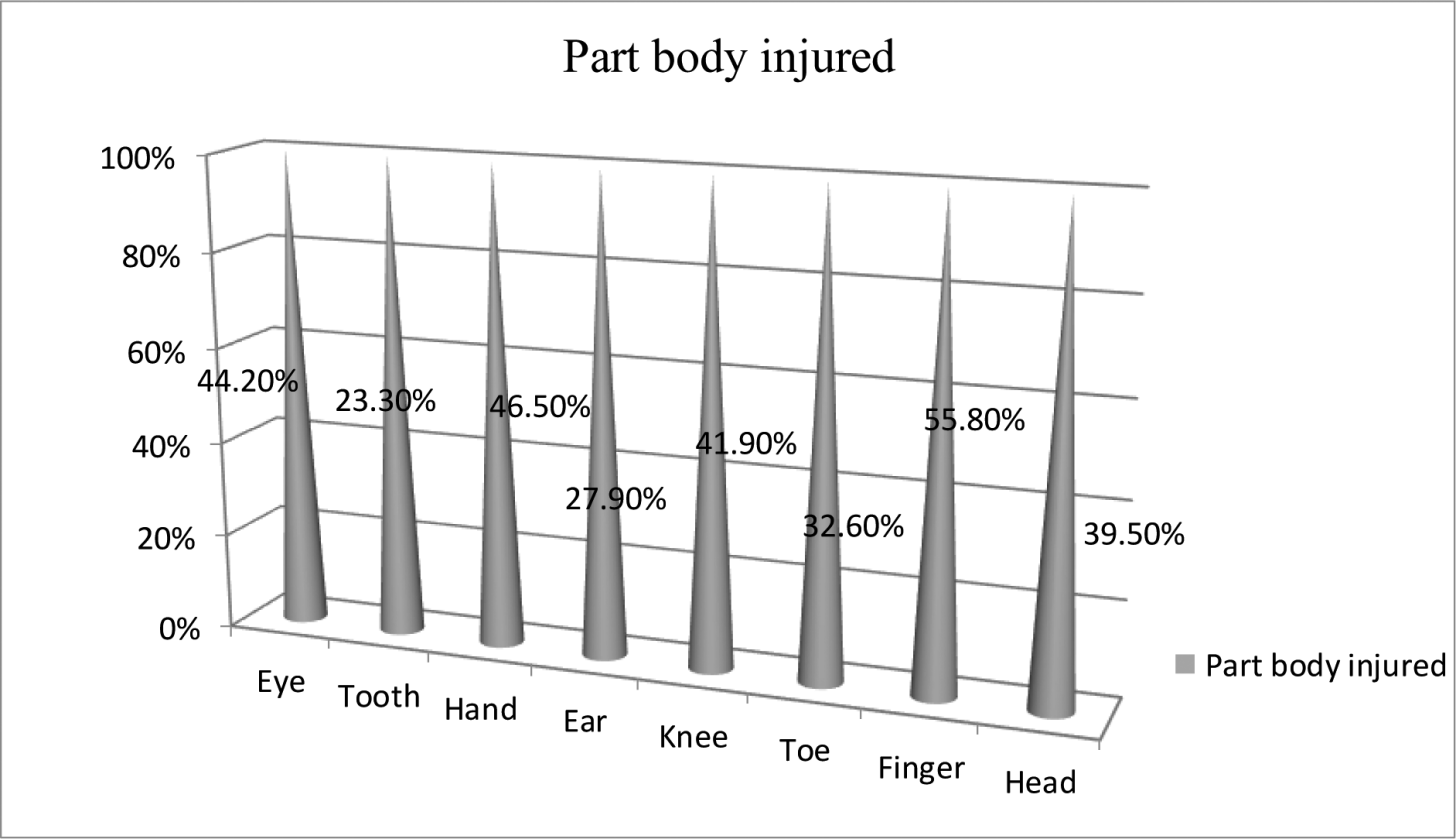
Parts of the body affected by occupational injury among Awash Wine Beverage Factory workers in Addis Ababa, Ethiopia, 2024.

Regarding the body part affected, 24 (55.8%) had trauma to the fingers, followed by the hand (20, 46.5%), while the tooth was the least affected body part (Fig. 2).

### 3.6. Working environment-related variables

Concerning the working environment, two-thirds of the participants (165, 60.9%) worked more than 40 hours. Two hundred one (74.2%) of workers had regular health and safety supervision, and 162 (59.8%) had safety training in connection with new employment, equipment, or work processes. One hundred two (74.5%) workers were involved in manual handling activities (pulling, pushing, carrying) with an average load of 6 to 25 kg. More than half of the participants (153; 56.3%) reported that their work required visual concentration, and 208 (76.8%) reported that their work does. Among those who use vibrating tools, 41 (26.8%) and 81 (52.9%) reported that the places where they worked were not always guarded or equipped with safety devices, and that the machines were not maintained immediately when old or unsafe, respectively (Table 4).

**Table 4:** Working environment-related characteristics of Awash Wine Beverage Factory workers in Addis Ababa, Ethiopia.

Variables	Category	Frequency	Percent
Hours worked per week	≤40 h	106	39.1
	>40 h	165	60.9
Regular health and safety supervision	No	70	25.8
	Yes	201	74.2
Had safety training in connection with new employment, equipment, and work process	No	109	40.2
	Yes	162	59.8
Work involves manual handling activity (pulling, pushing, carrying)	No	69	25.5
	Yes	202	74.5
Weight handled per day	light or <5 kg	82	40.6
	medium or 6–25 kg	89	44.1
	heavy or 25–50 kg	12	5.9
	Very heavy or >50 kg	22	10.9
Average time spent at work per day	<2 h	67	33.2
	2–4 h	111	55.0
	>4 h	27	13.4
The work needs visual concentration	No	63	23.2
	Yes	208	76.8
Using vibrating tools at your workplace	No	118	43.5
	Yes	153	56.5
For how long per day	<2 h	37	24.2
	2–4 h	103	67.3
	>4 h	13	8.5
The machines you are working with are always guarded or installed with a safety device	Yes	112	73.2
	No	41	26.8
Machines you are working with are always maintained immediately when old or	Yes	72	47.1
	No	81	52.9

### 3.7. Workers’ behavior and characteristics

**Figure 3.**
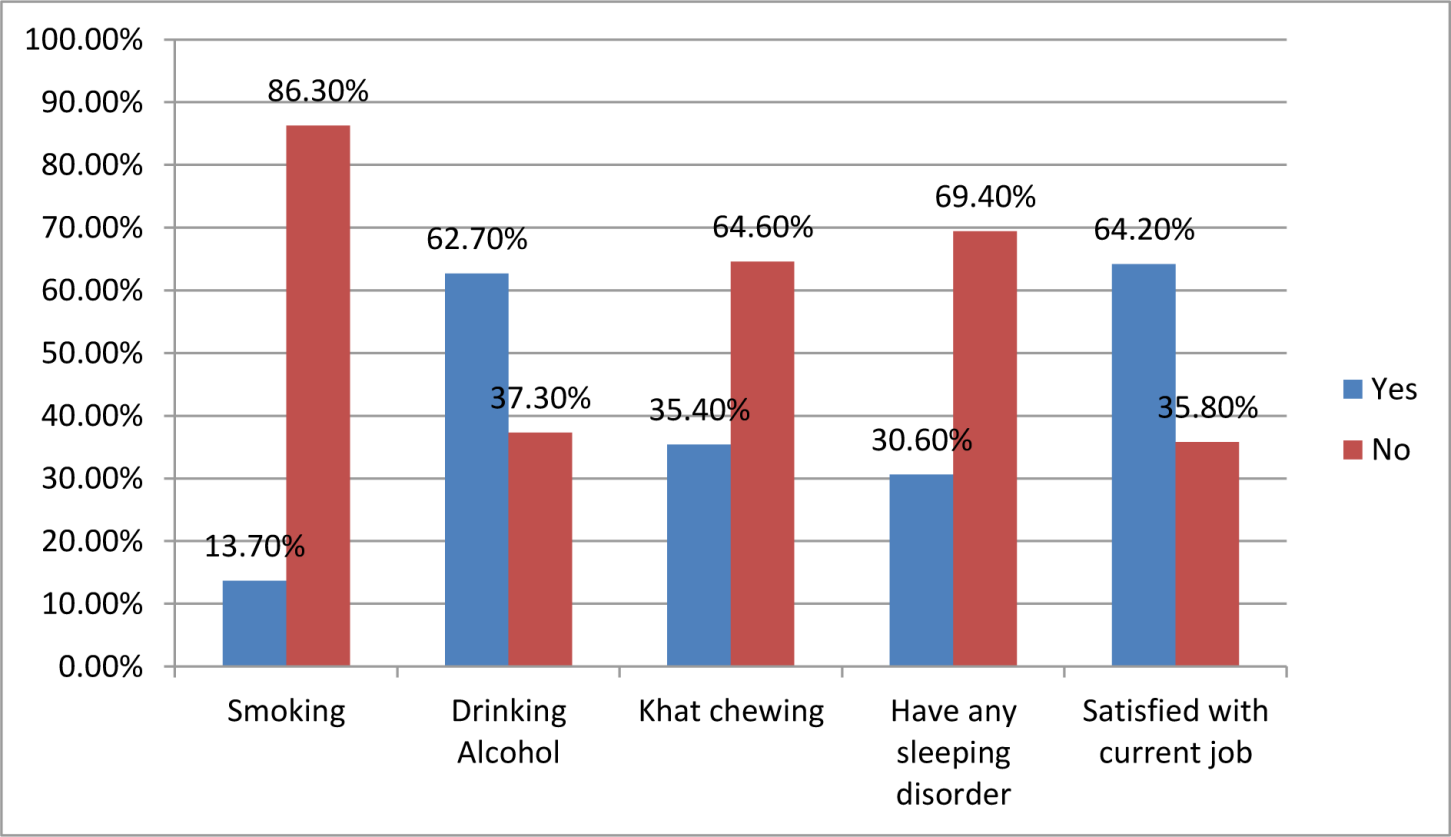
Workers’ behavior characteristics among Awash Wine Beverage Factory workers, in Addis Ababa, Ethiopia, 2024.

Regarding behavioral factors, 170 (62.7%) have a history of drinking alcohol, 37 (13.7%) of them had a history of smoking, 96 (35.4%) had a history of khat chewing, and 83 (30.6%) of participants reported having sleeping disorders; however, only 97 (35.8%) of them reported dissatisfaction with their current jobs (Fig. 3).

### 3.8. Knowledge about PPE practice on occupational injury

**Figure 4.**
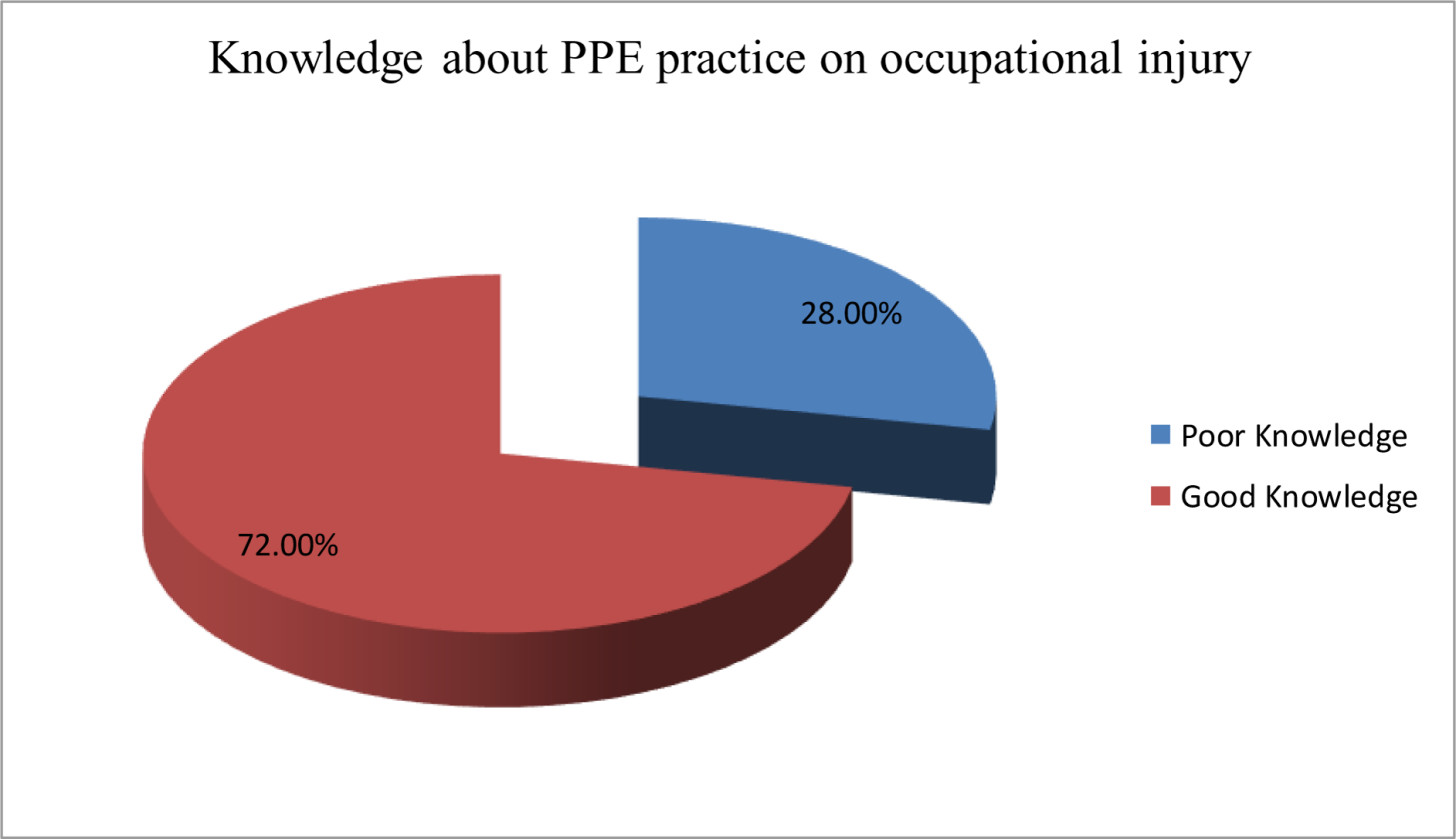
Knowledge about occupational injury among Awash Wine Beverage Factory workers in Addis Ababa, Ethiopia, 2024. PPE = personal protective equipment.

Among participants, 195 (72.0%) had good knowledge of occupational injuries, while the remaining 76 (28%) had poor knowledge (Fig. 4).

### 3.9. Factors associated with occupational injury

In the bivariate binary logistic regression model, variables such as sex, educational status, employment pattern, work experience, use of any PPE while at work, working condition, hours worked per week, regular health and safety supervision, having safety training, and knowledge passed the cutoff point for binary regression (*P*-value ≤ .25). These variables were included in the multivariable logistic regression model, and those with *P* ≤ .05 were considered significantly associated with occupational injury. Variables included in the multivariable model were selected a priori based on previous occupational health literature and theoretical relevance, including age, sex, work experience, and job category. Additional variables showing bivariate associations with the outcome were included to improve model stability.

Those workers with work experience ≤5 years were almost 6 times more likely to sustain an injury than those with >5 years of experience (adjusted odds ratio [AOR] = 5.86, 95% CI: 1.72–20.10). Compared with those who use PPE at work, not using PPE was associated with a 2.1-fold increase in the risk of injury (AOR = 2.06, 95% CI: 1.02–2.23). Moreover, workers at height were almost 8.7 times as likely to be exposed to occupational injury as those at ground level (AOR = 6.49, 95% CI: 5.94–8.85). Furthermore, those workers with no regular health and safety supervision were 5.9 times more likely to be exposed to occupational injury than those with proper supervision (AOR = 5.9, 95% CI: 1.77–19.62; Table 5).

**Table 5:** The bivariable and multivariable regression on occupational injury among Awash Wine Beverage Factory workers in Addis Ababa, Ethiopia.

Variables	Category	OI		COR (95% CI)	*P*-value	AOR (95% CI)	*P*-value
		Yes	No				
Sex	Male	30	127	1.84 (0.91–3.70)	.090	1.18 (0.38–3.94)	.777
	Female	13	101	1		1	
Educational status	Uneducated	19	85	1.73 (0.83–3.60)	.143	0.55 (0.16–1.87)	.340
	Educated	15	116	1		1	
Employment pattern	Temporary	13	42	1.92 (0.92–3.99)	.081	2.96 (0.86–10.21)	.086
	Permanent	30	186	1		1	
Work experience	<5 yr	27	83	2.95 (1.50–5.79)	.002	5.86 (1.72–20.10)*	**.005**
	≥5 yr	16	145	1		1	
Using any PPE while you are at work	No	17	39	3.17 (1.57–6.39)	.001	2.06 (1.02–2.23)*	**.000**
	Yes	26	189	1		1	
Working condition	At height	24	20	13.14 (6.16–28.0)	.000	8.69 (5.94–8.85)*	**.000**
	Ground and others	19	208	1		1	
Hours worked per week	≤40 h	14	123	1		1	
	>40 h	29	105	2.43 (1.22–4.83)	.012	1.70 (0.57–5.04)	.341
Regular health and safety supervision	No	20	50	3.10 (1.57–6.09)	.001	5.9 (1.77–19.62)*	.**004**
	Yes	23	178	1		1	
Had safety training	No	27	159	2.38 (1.23–4.62)	.010	2.70 (0.90–8.12)	.078
	Yes	16	69	1		1	
Knowledge	Poor knowledge	12	64	4.46 (2.14–9.30)	.000	2.54 (0.85–7.55)	.094
	Good knowledge	31	164	1		1	

Bold values represent variables that remained statistically significant in the multivariable logistic regression analysis and are reported with their AORs and 95% CIs.

AOR = adjusted odds ratio, CI = confidence interval, COR = crude odds ratio, OI = occupational injury, PPE = personal protective equipment.

*indicates variables that were statistically significant at a *P* < .05.

## 4. Discussion

The overall prevalence of occupational injury within 12 months before the study period was 43 (15.9%) with a 95% CI of 11.8% to 19.9%, while injuries 2 weeks before the study accounted for 14 (5.2%) with a 95% CI of 2.6% to 8.1%. Regarding the part of the body affected, 55.8% had trauma to the fingers, followed by the hand (46.5%). This study finding was higher than a study done on the analysis of causes, circumstances, and consequences of occupational traumatic injuries at food beverage enterprises in Ukraine from 2010 to 2018, which showed that in the food industry in beverage production, 221 workers were injured at the food factory within 8 years, averaging 27 workers injured within 12 months^[23]^ but lower than a study done in Harare, Zimbabwe, which revealed that 53.3% of participants reported having had a work-related injury. The most affected parts were the fingers (27.3%) and the hands (17.2%).^[24]^

A systematic review and meta-analysis on the prevalence of occupational injury and its associated factors in Ethiopia showed that the pooled prevalence of occupational injury in Ethiopia was 44.66%.^[14]^ Additionally, a study done on occupational injury and factors associated among employees in Heineken Brewery Share Company, Harar Brewery Share Company, and Awash Wine Share Company in Ethiopia revealed that the occupational injury rate was 20.9%, with fingers and hands being the most common type of trauma, and the prevalence of occupational injuries among beverage industry employees was high.^[9]^ The reasons might include methodological variations, the study’s scope, the study period, infrastructure differences, and differences in worker literacy.

Among factors associated with occupational injury within 12 months, work experience, PPE utilization, working conditions, and regular health and safety supervision were significant. Workers with ≤5 years of work experience were almost 5.9 times as likely to sustain an injury as those with >5 years of experience. However, my study contradicts studies conducted at Heineken Brewery Share Company and Harar Brewery Share Company, which found that 1 year of experience was protective.^[9]^ This might be because experienced workers are more knowledgeable about safety precautions and how to protect themselves from occupational injuries.

Compared with those who use any PPE while at work, those who do not use PPE were 2.1 times more likely to be injured. This study is supported by a systematic review in Ethiopia, which found that the odds of occupational injury were 3.01 times higher among individuals who did not use PPE than among those who did.^[15]^ Moreover, workers at height were almost 8.69 times as likely to sustain occupational injuries as those at ground level. Because the work involved working at height, the report showed that falling from height was the most common reason, reported by most participants.

The reason behind this might be that workers who do not use PPE lack essential protection against workplace hazards such as falling objects, sharp materials, and mechanical injuries. In addition, tasks performed at height increase the risk of loss of balance, slipping, or falling due to unstable surfaces, inadequate safety equipment, and limited protective barriers. These conditions substantially increase the likelihood of occupational injuries among workers.

Furthermore, workers with no regular health and safety supervision were 5.9 times as likely to be exposed to occupational injuries as those with proper supervision. This might be because professional supervisors may encourage workplace health by advising employees when they are in unsafe conditions. This study was supported by a systematic review conducted in Ethiopia that found the odds of occupational injury were 2.83 times higher among individuals with no health and safety supervision at work than among individuals with supervision.^[15]^

The reason might be that a lack of regular supervision can lead to poor adherence to safety guidelines, improper use of protective equipment, and failure to identify potential workplace hazards promptly. Supervisors play a critical role in monitoring workers’ practices, providing safety instructions, and enforcing OHS standards. Without such supervision, workers may engage in unsafe behaviors or overlook potential risks, thereby increasing their likelihood of experiencing occupational injuries.

## 5. Conclusion

This cross-sectional study identified that approximately 1 in 6 workers reported experiencing an occupational injury in the 12 months preceding the survey. Occupational injury was statistically associated with work experience, use of PPE, working conditions, and regular health and safety supervision. These findings describe observed associations at a single point in time and should not be interpreted as evidence of causality or risk prediction. The precision of some estimates should be interpreted cautiously, given the sample size and the potential for residual confounding. Despite these limitations, the results provide useful descriptive evidence on the distribution of occupational injuries and related workplace characteristics, highlighting areas that may warrant further investigation. Future studies employing longitudinal or experimental designs are needed to clarify temporal relationships and to strengthen the evidence base for occupational injury prevention strategies.

## 6. Limitations

The absence of injury logs or medical records and recall bias, especially for 12-month recall periods, were the study’s major limitations.

## Acknowledgments

The authors would like to express their gratitude to the health care professionals at the hospital for their generosity and invaluable assistance in collecting data and retrieving charts. Moreover, the authors wish to express their gratitude to the data collectors and supervisors.

## Author contributions

**Conceptualization:** Mequanente Dagnaw, Meera Indracanti.

**Data curation:** Mequanente Dagnaw, Meera Indracanti, Yordanos Kinfe, S. Hari Priya, Suleyman Mohammed Arage.
